# Is smokeless tobacco use associated with lower health-related quality of life? A cross-sectional survey among women in Bangladesh

**DOI:** 10.18332/tid/185969

**Published:** 2024-04-05

**Authors:** Rumana Huque, S. M. Abdullah, Sayem Ahmed, Nazmul Hossain, Farhin Islam, Mohammad A. B. Sarker, Md. Nurul Amin, Nasiruddin Ahmed

**Affiliations:** 1 Department of Economics, University of Dhaka, Dhaka, Bangladesh; 2 Research and Development, ARK Foundation, Dhaka, Bangladesh; 3 Department of Health Sciences, University of York, York, United Kingdom; 4 School of Health & Wellbeing, University of Glasgow, Glasgow, Scotland; 5 Health Economics Unit, Health Services Division, Ministry of Health and Family Welfare, Dhaka, Bangladesh

**Keywords:** Bangladesh, women, smokeless tobacco, EQ-5D-5L, Health-Related Quality of Life (HRQoL)

## Abstract

**INTRODUCTION:**

Bangladesh has 22 million adult users of smokeless tobacco (ST). The prevalence among women is higher (24.8%). Health-related quality of life outcome (HRQoL) for ST use is little known. We investigated the association between HRQoL and daily ST use among adult women in Bangladesh.

**METHODS:**

Using multi-stage design, a cross-sectional survey was conducted. Adult women (randomly selected) were surveyed from 4 purposively selected divisions (Dhaka, Chittagong, Khulna and Rangpur). Female ST users and non-users were compared using HRQoL scores. Self-perceived Visual Analogue Scale (EQ-VAS) values and HRQoL scores were modelled to examine their association with ST use.

**RESULTS:**

A total of 2610 women (1149 users and 1461 non-users) were surveyed. The proportion reported any type of problem in all health dimensions was significantly higher among female ST users than non-users (mobility: 43.3% vs 19.5%, self-care: 29.6% vs 11.9%, usual activities: 48.7% vs 21.8%, pain or discomfort: 69.8% vs 40.6%, and anxiety or depression: 61.3% vs 37.5%). The average HRQoL scores were 0.79 (95% CI: 0.78–0.81) and 0.90 (95% CI: 0.89–0.90) for users and non-users, respectively. Moreover, EQ-VAS average values were significantly higher for non-users [80.7 (95% CI: 79.9–81.6) vs 70.27 (95% CI: 69.2–71.2)]. Controlling the sociodemographics, ST use significantly reduced the HRQoL score by an average of 0.15 points. The EQ-VAS values on average decreased by 0.04 points for ST use.

**CONCLUSIONS:**

ST use is significantly associated with the HRQoL of females in Bangladesh. Considering the higher prevalence of ST, especially among women, HRQoL hazards need to be communicated for awareness building.

## INTRODUCTION

Bangladesh ranks second (after India), among 34 high smokeless tobacco (ST) burden countries^1,2^, with 22 million (20.6%) adult users^3^. ST products are popular, affordable, and socially acceptable in Bangladesh^4^. Unlike smoking tobacco, ST use is more prevalent among women (24.8%) than men (16.2%) in the country^3^. Low prices, increasing population, and misconceptions about health effects contribute to high ST use^2^. Though traditional values and social norms do not favor smoking by the young or by women, ST is embedded in spirituality, beliefs, festivals, lifestyle, and rituals such as marriage and popular entertainment^4^. Many deeply rooted myths and misconceptions are attached to ST use, especially in rural populations and among women^2^.

ST use presents a complex and widespread challenge to public health that has historically received little attention from policymakers in Bangladesh^5,6^. The ST products’ diversity and miscellaneous use patterns make the understanding of the impact complicated. These products are highly addictive and have several harmful consequences, therefore having no safer consumption level^7^. Being carcinogenic, the use of ST increases the risk of cancers of the oral cavity and pharynx^4,8-10^. In addition to risk factors for several dental problems such as cavities, abrasion of teeth, teeth staining, bad breath, gum disease, receding gums, bone loss around roots, and tooth loss, some forms of ST increase heart rate and blood pressure, which has implication for heart disease and stroke^4,10,11^. Furthermore, ST use imposes an economic burden on individuals^12^. However, research on ST-related mortality and morbidity in Bangladesh is scant^13^. Smoking-related mortality and morbidity research suggests that smoking is associated with lower Health-Related Quality of Life (HRQoL)^14-16^. However, across the countries, HRQoL outcomes from ST use are rarely known, although they are essential for evidence-informed policy decisions.

This study investigates the association between HRQoL and ST use among adult women in Bangladesh. HRQoL is widely used as a health status monitoring tool^17,18^. This has been used extensively for research on smoking^14-16^ and on a few occasions for ST^14,15^. Comparative evaluation of self-perceived and multidimensional HRQoL can reveal the health outcome of ST use. Research on the health impact of ST use has always been focused on pre-mature mortality and overlooked morbidity. Nevertheless, daily and intensive use of ST, especially among women, can have adverse implications for regular livelihood activities. The study aimed to bridge this knowledge gap by examining the association between ST use and HRQoL of women.

## METHODS

### Study design and sample size

The study was designed as a cross-sectional survey. Considering the area, population, and geographical diversity of Bangladesh, four divisions (Dhaka, Chittagong, Khulna, and Rangpur) were purposely selected for the survey. Since the ST prevalence is high among women, adult females aged ≥15 years^3^ were the participants. Applying division-wise ST prevalence^3^, the representative sample size was estimated as 2190 households (50% with female ST users and 50% with ST non-users) (Supplementary file Table 1A). Equal allocation was considered for the power and robustness of comparison. Considering a non-response of around 1%, 2213 households were surveyed.

### Sampling design and survey approach

A multi-stage sampling design was used. A total of 12 districts (three from each of the four divisions) were selected by simple random sampling. These were followed by subdistricts (called *upazilla*) and lower administrative units (called unions) under each subdistrict, which were selected randomly. The lowest administrative component, i.e. village or ward in each union, was the primary sampling unit (PSU). The households were randomly recruited in all the areas with urban–rural stratification. Estimated samples were equally distributed for all districts under each division (Supplementary file Table 2A).

*Household recruitment, characterization, and survey scope*

In a selected PSU, the sampling frame included all households with at least one adult female (self-reported) daily ST user (at least for the last year) as ‘user households’ and those with none of their members as users of any kind of tobacco as ‘non-user households’. In the user households, all the adult female ST users who were present during the survey were approached for participation. In contrast, in the non-user households, all available adult females were approached to take part. Since ST prevalence is predominant among women^3^ and smoking prevalence among them is negligible, the male respondents and smoking attributes were out of the survey scope.

### Survey tool, data collection, and storage

The survey was conducted simultaneously between October and November 2021 in four divisions. Written consent was obtained from the participants. The data were collected with a pre-tested semi-structured questionnaire, which the trained enumerators performed electronically with Survey CTO^19^. The questionnaire was designed following the Global Adult Tobacco Survey^3^. It had four sections: household characteristics and individual sociodemographic characteristics, ST use behavior and practices, onset of disease and treatment cost, and quality of life information. The quality-of-life segment was adopted from the EuroQol^17^. The translated Bengali version of the EQ-5D-5L questions was read out, and the enumerators recorded the responses in the Survey CTO application. For the EQ-VAS (Visual Analogue Scale), enumerators showed them the scale on a data collection device. They instructed the participants to assess their health state by selecting a point on the scale.

### Variables and data analysis

*Household and individual characteristics, ST use practice and disease onset*

The household characteristics such as age and gender of the household head, family size, location, and income and expenditure of the households, were collected. Sociodemographic characteristics of the individuals, such as age, gender, religion, marital status, occupation, and education level, were considered. Regarding ST use behavior and practice, at the individual level, daily used ST products, frequency of use, initiation age, influential person for initiation, quit attempt and quit duration, average expenditure behind ST, and source of the purchase, were enquired. The onset of diseases (cancer, asthma/COPD/breathing complications, heart disease, delivery complications, and oral diseases) for individuals was also considered. In practice, the women were asked to self-report the above disease onset and whether they were treated or under treatment.

*Quality of life measurement*

The quality of life was measured using EQ-5D instrument. This widely accepted, precise, and generic instrument for self-reported physical and mental health measures was introduced by EuroQol^17,20^. For descriptive health profiling, the EQ-5D-5L version was adopted, considering its less ceiling effects^20,21^. Besides, participants’ assessment of their general health state was coded through EQ-VAS^17^. Covering five health dimensions (mobility, usual activities, self-care, pain and discomfort, and anxiety and depression), quality of life was enquired with five levels centering around the extent of a problem: no, mild, moderate, severe, and extreme^20^. The level responses were recorded on a scale of 1 to 5 (1 = ‘no problem’ and 5 = ‘extreme problem’) while the EQ-VAS recorded the health state assessment on a scale from 0 (worst health one can think of) to 100 (best health one can aspire). Since EQ-5D value sets are unavailable for Bangladesh, Thailand’s value set (with the score ranging from -0.42 to 1.0) was referred for deriving the preference-based index value or HRQoL scores (also called EQ-5D-5L utility scores)^22^.

*Summary statistics and descriptive comparison*

Considering their sociodemographic characteristics, the EQ-5D-5L health state profiles of female ST users were compared with non-users. For the user group, the initiation age, influential person for ST uptake, product and daily frequency of use, quit attempt, and quit duration were analyzed. The difference in the proportion reporting ‘any sort of problem’ in health dimensions among female ST users and non-users, was tested statistically. Average HRQoL score and EQ-VAS values for the two groups were estimated concerning socioeconomic and sociodemographic factors, disease onset (cancer, oral disease, heart disease, delivery complications, asthma/COPD/breathing problem), ST use behavior and practice. The statistical significance of the difference in averages between the two groups, which were conditioning the mentioned factors, was tested. The correlation coefficient among them was measured, as a strong correlation between HRQoL score and EQ-VAS values is normally expected. Three different correlation (r) statuses were considered, defining them as: weak, r<0.3; moderate, 0.3≤r≤0.5; and strong, r>0.5^23^.

*Econometric modelling*

The impact of ST use on the outcome of HRQoL scores and EQ-VAS values was estimated using two different multiple linear regression models. In Model 1, the outcome was HRQoL scores, while in Model 2, it was EQ-VAS values. Both models had sociodemographic and ST-related exposures. The sociodemographic exposures were listed considering the significance of the difference in averages of HRQoL scores and EQ-VAS values for user and non-user females. A utility decrement or disutility score (1-HRQoL Score) was modeled to avoid negative HRQoL scores^23,24^. Thus, while exposure with a positive coefficient would increase the disutility score, it would decrease the HRQoL scores and worsen the health state^23^.

Since EQ-5D-5L data commonly have clustering effects, which leads to compromised consistency and biased estimates for ordinary least squares (OLS)^20^, a more robust estimation method, the Generalized Linear Model (GLM), was applied^25^. These are robust against non-normality and provide more unbiased and efficient estimates^23,26^. The GLM application method requires the identification of the ‘family of distribution’ and ‘link function for fitted values’^25^. An appropriate family of distribution (Gaussian, Gamma, Inverse Gamma, and Poisson) was chosen using the lowest χ^2^ value. Pregibon’s link test was used to decide the link function^23,27^. It was found that the disutility score followed a Poisson distribution, while EQ-VAS values followed the Gaussian distribution. The best link function for the fitted values of both the dependent variables was tested to be Log. Standard errors of the regression estimates were bootstrapped. STATA package *eq5dds* provided the descriptive profiling of HRQoL scores^28^. The value set was generated using the package named *eq5d* ^29^. All statistical calculations were operationalized using STATA 17^30^.

## RESULTS

### Socioeconomic statistics, disease onset, and ST use pattern

Around 86% of the ST users and 91% of non-user households had a male as their head. A higher number of female ST users resided in higher age groups (43% of female ST users aged ≥55 years). The female ST users mainly were married (71%), and slightly over a quarter were widows (27%). They also had low education level (63% had no schooling). The vast majority were homemakers (86%) (Supplementary file Table 3A). Most of the female ST users started at the age of 25–34 years (31%) or 15–24 years (30%). They were mainly influenced by in-laws (47%). It was found that around 6 to 7 users out of 10 used zarda or zarda with paan (betel leaf). A quarter used sada pata (sun-dried tobacco leaf) with paan, and gul (tobacco powder mixed with ingredients) was used by 13%, and 6% chewed sada pata directly. Around 4% used ST 6 to 10 times daily, while 8% used it more intensively (>15 times a day). Only 16% of users attempted to quit; among them, only 17% quit for a month or more (Supplementary file Table 4A).

The female ST users had higher disease (directly or indirectly attributed to ST use) onset than the non-users. Predominantly oral disease (teeth and/or gum problems) was more than twice as prevalent (27%) among the users than among the non-users (around 12%). Asthma/chronic obstructive pulmonary disease (COPD)/breathing problems, heart disease, and cancer (oral, laryngeal/esophageal/throat, lungs/chest, pancreas/stomach) were also more than twice as prevalent among the users (around 7.8%, 7.9%, and 1.3 %, respectively) (Supplementary file Figure 1A).

### Health outcome: application of EQ-5D-5L utility score

Regardless of health dimensions, a more significant proportion of female ST non-users reported no problems with their health state (mobility: 56.6% vs 80.4%, self-care: 70.3% vs 80.0%, usual activities: 51.2% vs 78.1%, pain or discomfort: 30.2% vs 59.3%, and anxiety or depression: 38.6% vs 62.4%) (Supplementary file Figure 2A). Table 1 contains the percentage of female ST users and non-users who had any type of problem (ranging from ‘slight’ to ‘extreme’) in all dimensions. Female users remained consistently higher in this regard than non-users. More specifically, considering movement, personal care, and usual activities, the percentage of women with ST use attributes facing any problem was more than double that of their non-user counterparts. For anxiety and depression, the percentages were 61.3% (95% CI: 58.5–64.1) and 37.5% (95% CI: 35.1–40.0) among users and non-users, respectively. A similar finding was observed for pain and discomfort [users: 69.8% (95% CI: 67.0–72.3) vs non-users: 40.6% (95% CI: 38.1–43.1)]. The differences in proportions were statistically significant.

**Table 1:** Percentage of ST user and non-user females aged ≥15 years who reported any problem (slight to extreme) in five dimensions of EQ-5D, October–November 2021 (N=2610)

*Health dimensions*	*ST user (N=1149)*			*ST non-user (N=1461)*			*p for differences in proportion*
	*n*	*%*	*95% CI*	*n*	*%*	*95 % CI*	
Movement	498	43.34	40.50–46.22	285	19.51	17.55–21.62	<0.001
Personal care	341	29.68	27.10–32.38	175	11.98	10.40–13.74	<0.001
Usual activities	560	48.74	45.85–51.63	319	21.83	19.78–24.02	<0.001
Pain and discomfort	802	69.80	67.07–72.38	594	40.66	38.16–43.19	<0.001
Anxiety and depression	705	61.36	58.50–64.13	549	37.58	35.12–40.09	<0.001

The overall minimum HRQoL score was -0.42. The average score for all women was 0.86, with a standard deviation (SD) of 0.19. The non-users had an average of 0.90 (95% CI: 0.89–0.90), while that for users was 0.79 (95% CI: 0.78–0.81). This difference was statistically significant at the 1% level (p<0.01). Also, for other socioeconomic and sociodemographic subgroups, the average HRQoL score for ST-using females was lower. Except for a few exceptions, the differences in average scores were statistically significant on all occasions (Table 2). Although the difference is small, the average utility score decreased for more intensive ST-using females. Those who used raw ST (e.g. chewing sada pata or ST with paan masala) had lower HRQoL scores.

**Table 2:** HRQoL average score and EQ-VAS average values of ST user and non-user females aged ≥15 years, October–November 2021 (N=2610)

*Characteristics*		*HRQoL average score*			*EQ-VAS average values*		
		*ST user (N=1149) Mean ± SE*	*ST non-user (N=1461) Mean ± SE*	*p*	*ST user (N=1149) Mean ± SE*	*ST non–user (N=1461) Mean ± SE*	*p*
**Distributional characteristics of index score of ST users and non-users**	Mean	0.79 ± 0.006	0.90 ± 0.004	<0.001	70.27 ± 0.523	80.76 ± 0.431	<0.001
	Median	0.83	0.94	-	75	85	-
	SD	0.20	0.16	<0.001	17.73	16.50	0.010
	Skewness	-1.98	-3.23	-	-0.61	-1.36	-
	Kurtosis	8.66	18.36	-	2.66	4.88	-
	Min, Max	(-0.30, 1)	(-0.42, 1)	-	(10, 100)	(5, 100)	-
**Distributional characteristics of overall index score**	Mean	0.86		-	76.14		-
	Median	0.90		-	80		-
	SD	0.19		-	17.83		-
	Skewness	-2.43		-	-0.93		-
	Kurtosis	11.50		-	3.35		-
	Min, Max	(-0.42, 1)		-	(5, 100)		-
**Household location**	Urban	0.83 ± 0.010	0.90 ± 0.011	<0.001	70.29 ± 1.311	81.15 ± 1.101	<0.001
	Rural	0.79 ± 0.006	0.90 ± 0.004	<0.001	69.94 ± 0.569	80.70 ± 0.468	<0.001
**Division**	Dhaka	0.83 ± 0.008	0.91 ± 0.008	<0.001	74.01 ± 0.973	83.45 ± 0.820	<0.001
	Chittagong	0.78 ± 0.011	0.90 ± 0.005	<0.001	72.30 ± 0.920	84.18 ± 0.631	<0.001
	Khulna	0.73 ± 0.016	0.86 ± 0.011	<0.001	68.81 ± 1.267	79.66 ± 1.043	<0.001
	Rangpur	0.84 ± 0.009	0.92 ± 0.006	<0.001	66.33 ± 0.921	75.54 ± 0.842	<0.001
**Marital status**	Married	0.83 ± 0.005	0.90 ± 0.004	<0.001	73.11 ± 0.570	80.78 ± 0.459	<0.001
	Otherwise	0.70 ± 0.014	0.87 ± 0.012	<0.001	63.34 ± 1.047	80.68 ± 1.142	<0.001
**Employment status**	Homemaker	0.81 ± 0.005	0.90 ± 0.004	<0.001	71.76 ± 0.534	80.39 ± 0.458	<0.001
	Otherwise employed	0.82 ± 0.017	0.87 ± 0.017	0.045	69.26 ± 1.850	75.45 ± 1.759	0.010
	Unemployed	0.53 ± 0.040	0.91 ± 0.018	<0.001	50.77 ± 2.266	87.05 ± 1.487	<0.001
**Education level**	No schooling	0.77 ± 0.008	0.81 ± 0.011	<0.001	67.69 ± 0.664	72.91 ± 0.995	<0.001
	Less than Primary	0.84 ± 0.013	0.88 ± 0.015	0.083	73.08 ± 1.317	75.08 ± 1.486	0.314
	Primary	0.84 ± 0.015	0.89 ± 0.009	0.003	74.80 ± 1.462	79.42 ± 1.022	0.010
	Less than secondary	0.86 ± 0.016	0.93 ± 0.004	<0.001	78.14 ± 1.426	85.38 ± 0.649	<0.001
	Secondary	0.82 ± 0.039	0.94 ± 0.009	0.005	76.36 ± 3.724	86.13 ± 1.058	0.018
	High school	0.74 ± 0.089	0.96 ± 0.007	0.050	66.42 ± 8.144	88.49 ± 1.091	0.035
	Graduate	0.94 ± 0.057	0.96 ± 0.012	0.770	72.50 ± 17.50	87.22 ± 2.212	0.553
**Household size**	1–3	0.81± 0.10	0.89 ± 0.007	<0.001	72.87 ± 0.948	80.10 ± 0.808	<0.001
	4–6	0.79 ± 0.007	0.90 ± 0.005	<0.001	69.48 ± 0.684	80.94 ± 0.542	<0.001
	≥7	0.77 ± 0.010	0.86 ± 0.018	<0.001	68.12 ± 1.506	81.84 ± 1.498	<0.001
**Wealth quintile**	Very low	0.80 ± 0.010	0.87 ± 0.011	<0.001	66.37 ± 1.148	75.82 ± 1.177	<0.001
	Low	0.84 ± 0.010	0.91 ± 0.008	<0.001	72.47 ± 1.051	79.90 ± 0.992	<0.001
	Medium	0.76 ± 0.016	0.90 ± 0.008	<0.001	70.87 ± 1.153	81.83 ± 0.840	<0.001
	High	0.79 ± 0.014	0.90 ± 0.009	<0.001	71.61 ± 1.140	80.82 ± 0.963	<0.001
	Very high	0.78 ± 0.015	0.89 ± 0.008	<0.001	70.78 ± 1.327	84.15 ± 0.858	<0.001
**Age** (years)	15–30	0.92 ± 0.009	0.95 ± 0.003	<0.001	82.04 ± 1.155	87.32 ± 0.453	<0.001
	31–45	0.87 ± 0.006	0.90 ± 0.004	<0.001	75.10 ± 0.843	79.13 ± 0.685	<0.001
	46–60	0.79 ± 0.008	0.79 ± 0.014	0.742	68.94 ± 0.819	69.41 ± 1.295	0.760
	61–75	0.67 ± 0.016	0.61 ± 0.042	0.185	61.52 ± 1.248	62.19 ± 2.892	0.831
	≥76	0.55 ± 0.048	0.52 ± 0.113	0.767	57.57 ± 2.722	64.81 ± 5.888	0.282
**Disease onset**	No disease	0.83 ± 0.007	0.92 ± 0.003	<0.001	74.06 ± 0.619	83.13 ± 0.433	<0.001
	Disease history	0.74 ± 0.010	0.82 ± 0.012	<0.001	64.98 ± 0.850	72.43 ± 1.090	<0.001
**Initiation age** (years)	≤15	0.77 ± 0.021	-	-	66.70 ± 1.748	-	-
	16–30	0.80 ± 0.008	-	-	70.99 ± 0.737	-	-
	31–45	0.82 ± 0.008	-	-	71.99 ± 0.907	-	-
	≥46	0.71 ± 0.022	-	-	66.18 ± 1.607	-	-
**Daily ST use intensity**	≤10	0.80 ± 0.006	-	-	71.24 ± 0.586	-	-
	11–20	0.77 ± 0.010	-	-	67.73 ± 1.191	-	-
	21–30	0.70 ± 0.048	-	-	64.80 ± 3.877	-	-
	≥31	0.78 ± 0.054	-	-	59.00 ± 5.610	-	-
**Quit attempt**	No	0.80 ± 0.006	-	-	71.35 ± 0.565	-	-
	Yes	0.76 ± 0.014	-	-	64.66 ± 1.297	-	-
**ST type**	Zarda and Paan	0.81 ± 0.006	-	-	71.23 ± 0.629	-	-
	Sada Pata and Paan	0.75 ± 0.013	-	-	69.59 ± 1.034	-	-
	Tobacco and Paan Masala	0.54 ± 0.013	-	-	48.75 ± 7.485	-	-
	Chewing Sada Pata	0.69 ± 0.034	-	-	63.05 ± 2.315	-	-
	Gul	0.78 ± 0.020	-	-	64.41 ± 1.620	-	-
	Khoini	0.72 ± 0.036	-	-	70.56 ± 2.522	-	-

SE: standard error. SD: standard deviation. Variance ratio tests for SD (ratio of SD of non-user group to user group is equal to 1).

The overall average of EQ-VAS was 76.14 (SD=17.83) with a median value of 80. The female ST non-users assessed their health status as being relatively well compared to the users [EQ-VAS averages were 80.76 (95% CI: 79.9–81.6) and 70.27 (95% CI: 69.2–71.2), for non-users and users, respectively]. Regardless of the sociodemographic characteristics, such a trend was persistent. Among the users who started using ST at a relatively younger age and used it more intensively had lower EQ-VAS average. The score was 71.24 for those who used ST less than 10 times daily. Conversely, it decreased to 59 for those who used it more than 30 times daily. Additionally, the EQ-VAS average was lower for those who used the raw form of ST (Table 2).

The distributions of HRQoL scores and EQ-VAS values had longer left wings and were more peaked than a normal distribution (Supplementary file Figure 3A). Additionally, the clustering effect of the scores was clearly visible. The participants were clustered around the value of 1 (perfect health) under the utility score. Although the clustering effect was not as severe as the utility score for the EQ-VAS values, participants were clustered around the scale range of 80–100.

The relationship between HRQoL scores and self-assessed EQ-VAS values was measured using a correlation coefficient along with its significance (Supplementary file Table 5A). In the overall sample, it was 0.63, while for the user and non-user subsets, they were 0.60 and 0.61, respectively. The association was statistically significant and categorized as strong. Except for a few occasions, this association for every subset of the data was strong and significant. Thus, irrespective of the sociodemographic characteristics and ST use status, females with higher HRQoL scores also had self-evaluated better health status in general.

### Econometric modelling: association of ST use with HRQoL scores and EQ-VAS values

Considering the non-normality and clustering of HRQoL scores, a GLM was estimated for disutility score with Poisson as the distribution family and Log as the link function. Results in Table 3 show that controlling all the sociodemographic factors, the average HRQoL score of female ST users was significantly lower by 0.15 points than that of the non-users. Furthermore, although the duration of use had no significant effect, intensive use of ST reduced scores significantly, with a coefficient value of 0.03 points. Area of residence, family size, and marital status were significant factors for the HRQoL score of females. In the test for appropriate distribution family, the χ^2^ for the Poisson distribution was found to be the lowest (0.06). The insignificance of the coefficient of the square of the predicted value of the base regression, established the appropriateness of the Log link in Pregibon’s link test (Supplementary file Table 6A).

**Table 3:** GLM estimation of disutility score (=1-HRQoL Score) with Poisson distribution and Log as link of ST user and non-user females aged ≥15 years, October–November 2021 (N=2610)

*Variables*		*Model 1 (Disutility as dependent)*	
		*Coeff.*	*p*
Residence (Ref. urban)	Rural	0.03	0.618
Family size	Number of family members	0.01	0.328
Respondent age	Age in years	0.09***	<0.001
	Square of age	-0.00***	<0.001
Family income	Monthly average	0.00*	0.070
Marital status (Ref. otherwise)	Married	0.06	0.243
Employment status (Ref. unemployed)	Homemaker	-0.45***	<0.001
	Otherwise employed	-0.32***	<0.001
Education level	Years of education	0.05**	0.012
	Square of education	-0.00***	<0.001
Disease history (Ref. none)	Have disease history	0.34***	<0.001
SLT status (Ref. non-user)	ST user	0.16*	0.087
Intensity of ST use	Number of times daily	0.03***	<0.001
	Square of intensity	-0.00**	0.039
Duration of ST use	Number of years using ST	-0.00	0.346
	Square of duration	0.00	0.464
Intercept	Constant	-4.95***	<0.001
Observations		2610	
Log likelihood		-877.50	
AIC (Akaike information criterion)		0.69	
BIC (Bayesian information criterion)		-20057.64	

Standard errors were bootstrapped.

* p<0.1,

** p<0.05,

*** p<0.01.

The GLM regression with EQ-VAS values as a response was estimated with a Gaussian distribution and Log as the link function (Table 4). It reinforced the findings from the HRQoL score regression. Controlling all the sociodemographics, the attribute of ST use among females decreased their EQ-VAS values. The coefficient was -0.04 (p<0.05). Although negative, the magnitude of the effect of duration of ST use was very small. Marital and education level and area of residence were significant contributing factors for EQ-VAS values. The value of the distribution family test was 14.94 for Gaussian distribution and the lowest compared to the others; additionally, the model did not fail Pregibon’s link test for Log as the link function (Supplementary file Table 7A).

**Table 4:** GLM estimation of EQ-VAS values with Gaussian distribution and Log as link of ST user and nonuser females aged ≥15 years, October–November 2021 (N=2610)

*Variables*		*Model 2 (EQ-VAS as dependent)*	
		*Coeff.*	*p*
Residence (Ref. urban)	Rural	-0.01	0.427
Family size	Number of family members	-0.01***	<0.001
Respondent age (years)	Age in years	-0.01***	<0.001
	Square of age	0.00	0.169
Family income	Monthly average	0.00***	<0.001
Marital status (Ref. otherwise)	Married	-0.02	0.219
Employment status (Ref. unemployed)	Homemaker	0.07***	<0.001
	Otherwise employed	0.02	0.357
Education level	Years of education	-0.00	0.807
	Square of education	0.00	0.185
Disease history (Ref. none)	Have disease history	-0.09***	<0.001
SLT use status (Ref. non-user)	ST user	-0.04**	0.042
Intensity of ST use	Number of times daily	-0.00	0.202
	Square of intensity	0.00	0.834
Duration of ST use	Number of years using ST	-0.00*	0.079
Intercept	Constant	4.64***	<0.001
Observations		2610	
Log likelihood		-10730.08	
AIC (Akaike information criterion)		8.23	
BIC (Bayesian information criterion)		548522.4	

Standard errors were bootstrapped.

* p<0.1,

** p<0.05,

*** p<0.01.

## DISCUSSION

The study examined the association between ST use and HRQoL outcomes among adult women in Bangladesh. The female ST users had a poor average HRQoL score compared to the non-users. EQ-5D-5L descriptive analysis showed that a larger number of female users reported problems in dimensions of HRQoL. Also, the self-assessed EQ-VAS average value was reportedly lower for them. Considering the several socioeconomic and sociodemographic factors, non-user females had a significantly higher average composite score of HRQoL and EQ-VAS values. Multivariate regression showed that controlling for socioeconomic and sociodemographic exposures, the presence of ST use, and its higher intensity, increased the disutility score and hence a negative factor for HRQoL score for females. The average score decreased by 0.15 points for female ST users. The findings remained the same when the association was examined with self-assessed EQ-VAS values. Earlier studies have also found a similar association between HRQoL outcome and ST use and concluded about the adverse implication of the latter on the former^14,15^.

Previous studies reported differences in EQ-5D-5L scores by age, gender, and smoking status^31,32^. It was found that currently smoking women in those aged 45–54 years were expected to have an EQ-5D-5L score of 0.89 compared to 0.92 for women in the same age group who had never smoked in Spain^32^. The scores were higher among men for the same age group. A recent study in China suggested that smokers and non-smokers had an EQ-5D index of 0.82 (SD=0.14) and 0.80 (SD=0.13), respectively^31^. The EQ-VAS score was found to be statistically different among smokers (mean=77.3, SD=21.9) and non-smokers (mean=84.4, SD=14.8) (all p<0.001)^31^. Our estimated HRQoL score of female ST users was close to the score of current smokers in Spain and China. Further studies are required to estimate the gender and age-disaggregated HRQoL in Bangladesh.

The research findings related to the disease onset reinforced the existing evidence. The female users of ST reportedly had a twice larger extent of oral disease, heart disease, asthma, and COPD than non-user females. The examination of ST use patterns and behavior revealed that ST use is associated with age, education level, and socioeconomic status. The ST use rate increases consistently with age, lower level of education, and lower household income. This finding is in line with previous research related to ST use prevalence and its health impact^1,4,6,33-35^. Hence, an extensive cessation program with special focus on ST use is warranted for controlling the prevalence of tobacco in Bangladesh. The inverse relationship between socioeconomic status and ST use prevalence and the gender perspective, highlight the importance of developing different tobacco control strategies for women. As the use of ST is culturally accepted in Bangladesh, culturally appropriate public awareness campaigns are required to curb it^4^. Public awareness strategies regarding the health hazards of ST use should be implemented at the household level, and social movement needs to be in place to combat cultural acceptance since the prevalence of ST use is predominantly higher among women while users are mostly homemakers; also, community-based and individual-level intervention should be delivered to the target population. Aside from various social interventions, economic and governance interventions also need to be implemented. Appropriate price and tax measures should be adopted to make ST products unaffordable^6^.

### Limitations

The study has some limitations. The non-users were identified through self-report. No biomarker was used to verify their ST use status. However, as ST use is socially and culturally acceptable, we believe that there was less incentive for the user to hide their consumption status. Although the study sample is representative at the divisional level, the generalizability of the findings is limited as it used only four divisions. The external validity of the findings is also limited for other countries. Additionally, since the findings used cross-sectional data instead of cohort analysis, the effect measure might not be a true causal impact. The residual confounding was not controlled for in the multivariate models. Despite the shortcomings, the study fills the research gap by underlining the evidence that ST use has severe implications for the quality functioning of life of women in Bangladesh.

## CONCLUSIONS

ST use is significantly associated with women’s HRQoL. The analysis of EQ-5D-5L data reveals that women who did not consume ST had improved HRQoL and, hence, had self-perceived better physical and mental health. Therefore, considering the higher prevalence of ST, especially among women, ST control policies should be prioritized. Social interventions communicating the HRQoL hazards can be activated for awareness building and to achieve control over this socially accepted tobacco.

## Supplementary Material



## Data Availability

The data supporting this research are available from the authors on reasonable request.

## References

[1] Sinha DN, Rizwan SA, Aryal KK, Karki KB, Zaman MM, Gupta PC. Trends of Smokeless Tobacco use among Adults (Aged 15-49 Years) in Bangladesh, India and Nepal. Asian Pac J Cancer Prev. 2015;16(15):6561-6568. doi:10.7314/apjcp.2015.16.15.6561 26434875

[2] Ahmed N. Economics of Smokeless Tobacco Taxation in Bangladesh. Tob. Prev. Cessation. 2019;(suppl. 5):Α117. doi:10.18332/tpc/105199

[3] Bangladesh Bureau of Statistics; National Tobacco Control Cell; World Health Organization. Global Adult Tobacco Survey (GATS). Bangladesh Bureau of Statistics and National Tobacco Control Cell; 2019. Accessed March 14, 2024. https://ntcc.gov.bd/ntcc/uploads/editor/files/GATS%20Report%20Final-2017_20%20MB.PDF

[4] Huque R, Zaman MM, Huq SM, Sinha DN. Smokeless tobacco and public health in Bangladesh. Indian J Public Health. 2017;61(suppl 1):S18-S24. doi:10.4103/ijph.IJPH_233_17 28928314 PMC6349136

[5] Khan A, Huque R, Shah SK, et al. Smokeless tobacco control policies in South Asia: a gap analysis and recommendations. Nicotine Tob Res. 2014;16(6):890-894. doi:10.1093/ntr/ntu020 24616238

[6] Huque R, Al Azdi Z, Sheikh A, et al. Policy priorities for strengthening smokeless tobacco control in Bangladesh: a mixed-methods analysis. Tob Induc Dis. 2021;19(October):78. doi:10.18332/tid/140826 34707471 PMC8500203

[7] Huque R, Shah S, Mushtaq N, Siddiqi K. Determinants of salivary cotinine among Smokeless Tobacco users: a cross-sectional survey in Bangladesh. PLoS One. 2016;11(8):e0160211. doi:10.1371/journal.pone.0160211 27504912 PMC4978394

[8] Boffetta P, Hecht S, Gray N, Gupta P, Straif K. Smokeless tobacco and cancer. Lancet Oncol. 2008;9(7):667-675. doi:10.1016/S1470-2045(08)70173-6 18598931

[9] Zaman MM, Ahmed J, Choudhury SR, Numan SM, Parvin K, Islam MS. Prevalence of ischemic heart disease in a rural population of Bangladesh. Indian Heart J. 2007;59(3):239-241. 19124932

[10] Hajat C, Stein E, Ramstrom L, Shantikumar S, Polosa R. The health impact of smokeless tobacco products: a systematic review. Harm Reduct J. 2021;18(1):123. doi:10.1186/s12954-021-00557-6 34863207 PMC8643012

[11] Piano MR, Benowitz NL, Fitzgerald GA, et al. Impact of smokeless tobacco products on cardiovascular disease: implications for policy, prevention, and treatment: a policy statement from the American Heart Association. Circulation. 2010;122(15):1520-1544. doi:10.1161/CIR.0b013e3181f432c3 20837898

[12] John RM, Sinha P, Munish VG, Tullu FT. Economic costs of diseases and deaths attributable to tobacco use in India, 2017-2018. Nicotine Tob Res. 2021;23(2):294-301. doi:10.1093/ntr/ntaa154 32805055

[13] Zaman MM, Bhuiyan MR, Karim MN, et al. Clustering of non-communicable diseases risk factors in Bangladeshi adults: an analysis of STEPS survey 2013. BMC Public Health. 2015;15:659. doi:10.1186/s12889-015-1938-4 26169788 PMC4501055

[14] Xu X, Fiacco L, Rostron B, et al. Assessing quality-adjusted years of life lost associated with exclusive cigarette smoking and smokeless tobacco use. Prev Med. 2021;150:106707. doi:10.1016/j.ypmed.2021.106707 34186150

[15] Ahsan I, Menon I, Gupta R, Arora V, Ashraf A, Das D. Oral health-related quality of life among adult smokeless tobacco users and nontobacco users of Ghaziabad district, Uttar Pradesh: a cross-sectional study. Journal of Indian Association of Public Health Dentistry. 2021;19(1):48-54. doi:10.4103/jiaphd.jiaphd_60_20

[16] Sagtani RA, Thapa S, Sagtani A. Smoking and quality of life - Is there really an association? Evidence from a Nepalese sample. PLoS One. 2019;14(9):e0221799. doi:10.1371/journal.pone.0221799 31490943 PMC6730920

[17] EuroQol Research Foundation. EQ-5D-5L User Guide Version 3.0 September 2019; 2019. Accessed March 14, 2024. https://euroqol.org/publications/user-guides

[18] Ravens-Sieberer U. Measuring and monitoring quality-of-life in population surveys: still a challenge for public health research. Soz Praventivmed. 2002;47(4):203-204. doi:10.1007/BF01326397 12415920

[19] Dobility, Inc. SurveyCTO; 2017. Accessed March 14, 2024. https://www.surveycto.com/

[20] Devlin N, Parkin D, Janssen B. Methods for Analysing and Reporting EQ-5D Data. Cham (CH): Springer; 2020. doi:10.1007/978-3-030-47622-9 33347096

[21] Janssen MF, Birnie E, Bonsel GJ. Quantification of the level descriptors for the standard EQ-5D three-level system and a five-level version according to two methods. Qual Life Res. 2008;17(3):463-473. doi:10.1007/s11136-008-9318-5 18320352 PMC2275305

[22] Pattanaphesaj J, Thavorncharoensap M, Ramos-Goñi JM, Tongsiri S, Ingsrisawang L, Teerawattananon Y. The EQ-5D-5L Valuation study in Thailand. Expert Rev Pharmacoecon Outcomes Res. 2018;18(5):551-558. doi:10.1080/14737167.2018.1494574 29958008

[23] Nguyen LH, Tran BX, Hoang Le QN, Tran TT, Latkin CA. Quality of life profile of general Vietnamese population using EQ-5D-5L. Health Qual Life Outcomes. 2017;15(1):199. doi:10.1186/s12955-017-0771-0 29020996 PMC5637080

[24] Emrani Z, Akbari Sari A, Zeraati H, Olyaeemanesh A, Daroudi R. Health-related quality of life measured using the EQ-5D-5 L: population norms for the capital of Iran. Health Qual Life Outcomes. 2020;18(1):108. doi:10.1186/s12955-020-01365-5 PMC7183694 32334604

[25] Nelder JA, Wedderburn RWM. Generalized linear models. J R Stat Soc Ser A. 1972;135(3):370–84. doi:10.2307/2344614

[26] Mihaylova B, Briggs A, O’Hagan A, Thompson SG. Review of statistical methods for analysing healthcare resources and costs. Health Econ. 2011;20(8):897-916. doi:10.1002/hec.1653 20799344 PMC3470917

[27] Pregibon D. Link Tests. Encyclopedia of Statistical Sciences. Wiley; 2004. doi:10.1002/0471667196.ess1479

[28] Ramos-Goñi JM, Ramallo-Fariña Y. Eq5dds: A command to analyze the descriptive system of EQ-5D Quality-of-life Instrument. Stata J. 2016;16(3):691–701. doi:10.1177/1536867X1601600309

[29] Ramos-Goñi JM, Rivero-Arias O. Eq5d: A command to calculate Index Values for the EQ-5D Quality-of-life Instrument. Stata J. 2011;11(1):120–125. doi:10.1177/1536867X1101100108

[30] StataCorp LLC. Stata Statistical Software: Release 17. 2021. College Station, Texas, United States of America.

[31] Hu K, Zou S, Zhang CJ, et al. Health-related quality of life among pregnant women with pre-pregnancy smoking and smoking cessation during pregnancy in China: National cross-sectional study. JMIR Public Health Surveill. 2022;8(1):e29718. doi:10.2196/29718 35072649 PMC8822427

[32] López-Nicolás Á, Trapero-Bertran M, Muñoz C. Smoking, health-related quality of life and economic evaluation. Eur J Health Econ. 2018;19(5):747-756. doi:10.1007/s10198-017-0919-1 28748308

[33] Choudhury K, Hanifi SM, Mahmood SS, Bhuiya A. Sociodemographic characteristics of tobacco consumers in a rural area of Bangladesh. J Health Popul Nutr. 2007;25(4):456-464. 18402189 PMC2754020

[34] Ashraf A, Quaiyum MA, Ng N, et al. Self-reported use of tobacco products in nine rural INDEPTH Health and Demographic Surveillance Systems in Asia. Glob Health Action. 2009;2:10.3402/gha.v2i0.1997. doi:10.3402/gha.v2i0.1997 PMC2785137 20027256

[35] Inamdar AS, Croucher RE, Chokhandre MK, Mashyakhy MH, Marinho VC. Maternal smokeless tobacco use in pregnancy and adverse health outcomes in newborns: a systematic review. Nicotine Tob Res. 2015;17(9):1058-1066. doi:10.1093/ntr/ntu255 25534929

