# Supplementary material for: Is smokeless tobacco use associated with lower health-related quality of life? A cross-sectional survey among women in Bangladesh
Source: Tob Induc Dis. 2024 Apr 5;22:10.18332/tid/185969. doi: 10.18332/tid/185969 (PMC10996035; doi:10.18332/tid/185969)
Supplement: Supplementary file 1 [file TID-22-60-s1.pdf]

Table 1A: ST Prevalence and Estimated Sample Size by Division

Division	ST Prevalence (%) (GATS 2017)	Estimated Sample Size (Households)	Sample Approximation (Households)
Chittagong	19.9	245	255
Dhaka	22.4	267	270
Khulna	22.8	270	270
Rangpur	25.6	293	300
**Total**		**997**	**1,095**

Note: The sample approximation is done in a way that equalizes the distribution of the sample in each randomly selected district under a division. For example, under Chittagong division three randomly selected districts were Chattogram, Brahmanbaria, and Bandarban. The estimated sample size for this division was 245 the optimum sample approximation was 255 so that each of the three districts had an equal number of 85 households included in the survey.

Table 2A: Sampling Design and Study Areas

Division	Districts	Upazila	Union	Village/Word (Primary Sampling Unit - PSU)	Survey Sample of HH with SLT Using Women Member		
					District Total (User HH)	District Total (Non User HH)	Divisional Total
**Chittagong**	Chattogram	Anowara	Boirag	1. Uttar Gua Panchak	85	85	510
				2. Dakkhin Gua Panchak			
			Chaturi	1. Belchura			
				2. Yakub			
				3. Chairmanbari			
	Brahmanbaria	Ashugonj	Haraishidha	1. Haraishidha	85	85	
				2. Bhobanipur			
	Bandarban	Lama	Lama Sadar	1. Sabekbilchari	85	85	
				2. Marma Para			
				3. Chagalkhaiya			
			Ruposhipara	1. Angholapara			
				2. Chairmenpara			
				3. Karbaripara			
**Dhaka**	Dhaka	Bhashantek	Ward	1. Ward 4	90	90	540
		Pollobi		2. Ward 2,4,7			
		Lalbag		3. Ward 43			
	Manikganj	Shingair	Jhikutia	1. Horirampur	90	90	
	Tangail	Delduar	Atia	1. Kosba Atia	90	90	
				2. Kandapara			
				3. Gomjani			
**Khulna**	Khulna	Fulltola	Atra Gilatola	1. Gabtola	90	90	540
				2. Mushiyali			
				3. Esterngate			
			Damudar	1. Damudar			
				2. Pother Bazar			
	Satkhira	Tala	Jathpur	1. Ghona	90	90	
				2. Hajarikathi			
				3. Jathpur			
	Kushtia	Kumarkhali	Nandolalpur	1. Nandolalpur	90	90	
			Koya	1. Koya			
**Rangpur**	Lalmonirhat	Kaligonj (Lalmonirhat)	Tushvander	1. Kashiram	100	100	600
				2. Boirati			
	Kurigram	Kurigram Sadar (Kurigram)	Pachgachi	1. Panaki Para	100	100	
				2. Masterpara			
			Jatrapur	1. Mondolpara			
				2. Shyampur			
	Dinajpur	Dinajpur Sadar	Balubari	1. Khalpara	100	100	
				2. Nimtuli			
			Fulbari	1. Collegepara			
				2. Methorpotti			
**Total: 4 Divisions**	**12 Districts**				**1,095**	**1,095**	**2,190**

Table 3A: Household and Individual Characteristics

	Categories	User		Non-user		Overall	
		n	%	n	%	n	%
**Household (HH) Characteristics**							
Sample	User HH	-	-	-	-	1106	49.98
	Non-user HH	-	-	-	-	1107	50.02
Sex of HH Head	Male	952	86.08	1008	91.06	1960	88.57
	Female	154	13.92	99	8.94	253	11.43
Age of HH Head	15 - 24	13	1.18	36	3.25	49	2.21
	25 - 34	138	12.48	229	20.69	367	16.58
	35 - 44	251	22.69	355	32.07	606	27.38
	45 - 54	234	21.16	240	21.68	474	21.42
	55 and Above	470	42.50	247	22.31	717	32.40
HH Size	01 - 03	324	29.29	410	37.04	734	33.17
	04 - 06	650	58.77	649	58.63	1299	58.70
	07 and Above	132	11.93	48	4.34	180	8.13
Number of Children	0	371	33.54	264	23.85	635	28.69
	1	339	30.65	400	36.13	739	33.39
	2	283	25.59	320	28.91	603	27.25
	3 and Above	113	10.22	123	11.11	236	10.66
Monthly Income (Last 12 Month)	10,000 and Below	434	39.24	394	35.59	828	37.42
	10,001 - 20,000	481	43.49	509	45.98	990	44.74
	20, 001 - 30,000	137	12.39	135	12.20	272	12.29
	30,001 - 40,000	34	3.07	44	3.97	78	3.52
	Above 40,000	20	1.81	25	2.26	45	2.03
Monthly Expenditure (Last 12 Month)	10,000 and Below	502	45.39	503	45.44	1005	45.41
	10,001 - 20,000	504	45.57	507	45.80	1011	45.68
	20, 001 - 30,000	87	7.87	74	6.68	161	7.28
	30,001 - 40,000	8	0.72	16	1.45	24	1.08
	Above 40,000	5	0.45	7	0.63	12	0.54
**Individual Characteristics**							
Age of Individuals	15 - 24	27	2.35	367	25.12	394	15.1
	25 - 34	128	11.14	413	28.27	541	20.73
	35 - 44	220	19.15	348	23.82	568	21.76
	45 - 54	283	24.63	173	11.84	456	17.47
	55 and Above	491	42.73	160	10.95	651	24.94
Educational Attainment	No Schooling	736	64.06	343	23.48	1079	41.34
	< Primary	167	14.53	143	9.79	310	11.88
	Primary	116	10.1	228	15.61	344	13.18
	< Secondary	98	8.53	466	31.9	564	21.61
	Secondary	22	1.91	171	11.7	193	7.39
	High School	7	0.61	77	5.27	84	3.22
	Graduation	2	0.17	22	1.51	24	0.92
	Post Graduation	1	0.09	11	0.75	12	0.46
Maritial Status	Married	815	70.93	1176	80.49	1991	76.28
	Unmarried	2	0.17	149	10.2	151	5.79
	Widow	308	26.81	121	8.28	429	16.44
	Separated, Divorced	24	2.09	15	1.03	39	1.49
Occupation	Govt. Employee	0	0	11	0.75	11	0.42
	Non Govt. Employee	3	0.26	14	0.96	17	0.65
	Business (Small)	6	0.52	4	0.27	10	0.38
	Business (Large)	0	0	1	0.07	1	0.04
	Farming	2	0.17	0	0	2	0.08
	Industrial Worker	5	0.44	5	0.34	10	0.38
	Daily Labourer	33	2.87	32	2.19	65	2.49
	Self Employed	8	0.7	7	0.48	15	0.57
	Student	2	0.17	131	8.97	133	5.1
	Housemaid/Housework	31	2.7	22	1.51	53	2.03
	Retired	5	0.44	1	0.07	6	0.23
	Unemployed	45	3.92	18	1.23	63	2.41
	Homemaker	990	86.24	1212	82.96	2202	84.4
	Other	18	1.57	3	0.21	21	0.8

Table 4A: ST Using Practice

Characteristics	Categories	Frequency (N)	Percent (%)
Initiation Age	05 - 14	60	5.22
	15 - 24	348	30.29
	25 - 34	354	30.81
	35 - 44	231	20.10
	45 and Above	156	13.58
Influential Person	Nobody	264	22.98
	Family Members	251	21.85
	In-laws	535	46.56
	Friends and Colleagues	59	5.13
	Others	40	3.48
Daily Used ST Products	Zarda / Zarda with Paan	758	65.97
	Sada Pata with Paan	291	25.33
	Tobacco with Paan Masala	8	0.70
	Chewing Sada Pata	68	5.92
	Gul	148	12.88
	Khoini	53	4.61
	Others	1	0.09
Daily Frequency of Use	1 - 5	416	36.21
	6 - 10	472	41.08
	11 - 15	172	14.97
	16 and Above	89	7.75
Quit Attempt	Yes	186	16.19
	No	963	83.81
Quit Duration	1-7 Days	96	51.61
	8-30 Days	58	31.18
	>30 Days	32	17.20

Table 5A: Correlation of HRQoL Scores and EQ-VAS Values

Characteristics	Category	N	Correlation between EQ – 5D – 5L Index and EQ-VAS Score	Significance of Correlation Coefficient (P – Value)
Overall		2610	0.63***	<0.001
ST Using Status	User	1149	0.60***	<0.001
	Non - User	1461	0.61***	<0.001
HH Location	Urban	160	0.56***	<0.001
	Rural	2256	0.65***	<0.001
Division	Dhaka	644	0.59***	<0.001
	Chittagong	648	0.73***	<0.001
	Khulna	660	0.65***	<0.001
	Rangpur	658	0.69***	<0.001
Marital Status	Married	1991	0.58***	<0.001
	Otherwise	619	0.70***	<0.001
Employment Status	Homemaker	2202	0.59***	<0.001
	Otherwise Employed	184	0.69***	<0.001
	Unemployed	224	0.80***	<0.001
Educational Attainment	No Schooling	1079	0.59***	<0.001
	< Primary	310	0.56***	<0.001
	Primary	344	0.64***	<0.001
	< Secondary	564	0.60***	<0.001
	Secondary	193	0.51***	<0.001
	High School	84	0.60***	<0.001
	Graduation	7	0.65***	<0.001
	Post-Graduation	12	0.75***	<0.001
HH Size	01 - 03	773	0.62***	<0.001
	04 - 06	1562	0.63***	<0.001
	07 and Above	275	0.66***	<0.001
Wealth Quintile	Very Low	523	0.57***	<0.001
	Low	521	0.59***	<0.001
	Medium	538	0.67***	<0.001
	High	525	0.67***	<0.001
	Very High	503	0.70***	<0.001
Age Group	15 – 30	788	0.58***	<0.001
	31 – 45	868	0.48***	<0.001
	46 – 60	620	0.50***	<0.001
	61 – 75	276	0.58***	<0.001
	76 and Above	58	0.72***	<0.001
Disease Event	No Disease	1807	0.61***	<0.001
	Have Disease History	803	0.58***	<0.001
Initiation Age	≤15	131	0.64***	<0.001
	16 – 30	592	0.62***	<0.001
	31 – 45	307	0.53***	<0.001
	≥46	119	0.57***	<0.001
Daily ST Using Intensity	≤10	889	0.60***	<0.001
	11 - 20	219	0.63***	<0.001
	21 - 30	26	0.66***	<0.001
	≥31	15	0.30	0.280
Quit Attempt	No	963	0.62***	<0.001
	Yes	186	0.47***	<0.001
ST Type	Zarda & Paan	758	0.64***	<0.001
	Sada Pata & Paan	291	0.60***	<0.001
	Tobacco & Paan Masala	8	0.67*	0.070
	Chewing Sada Pata	68	0.72***	<0.001
	Gul	148	0.52***	<0.001
	Khoini	53	0.73***	<0.001

Note: ***, ** and * indicates significance at 1%, 5% and 10% level respectively.

Testing the GLM Family of Distribution and Link Function for HRQoL Score Regression

Table 6A: GLM with Gamma as Distribution and Log as Link

Variables		Model 1 (Disutility as Dependent)	
		**Coeff.**	**P - Value**
Residence (*Ref:* Urban)	Rural	-0.00	0.972
Family Size	Number of Family Members	0.02	0.413
Respondent Age	Age in Years	0.10^{***}	<0.001
	Age Square	-0.00^{***}	<0.001
Family Income	Monthly Average	0.00	0.417
Marital Status (*Ref:* Otherwise)	Married	0.07	0.460
Employment Status (*Ref:* Unemployed)	Homemaker	-0.43^{***}	<0.001
	Otherwise Employed	-0.18	0.286
Educational Attainment	Years of Education	0.02	0.332
	Square of Education	-0.00^{**}	0.044
Disease History (*Ref:* None)	Have Disease History	0.46^{***}	<0.001
ST Status (*Ref:* Non-User)	ST User	0.01	0.940
Intensity of ST Use	Number of Times Daily	0.03^{*}	0.077
	Intensity Square	-0.00	0.210
Duration of ST Use	Number of Years using ST	-0.01	0.136
	Duration Square	0.00	0.209
Intercept	Constant	-5.07^{***}	<0.001
Observations		2610	
Log likelihood		3295.75	
AIC		-2.51	
BIC		-19026.6	

Test Regression

Dependent Variable: rawvar = ((disutility - rawyhat) ^{2})	Coeff.	P - Value
xbeta1 (Predicted Value of First Regression)	0.96^{***}	<0.001
Intercept	-1.94^{***}	<0.001
Log likelihood	8126.68	
AIC	-6.23	
BIC	-12993.28	

Testing Distribution Family

Hypothesis	Chi - Square	Prob.	Rule: If Do not Reject the Hypothesis then	Decision
Coefficient of xbeta1 = 0	38.98	0.000	Family is Gaussian	Not Gaussian
Coefficient of xbeta1 = 1	0.06	0.804	Family is Poission	Poission Family
Coefficient of xbeta1 = 2	45.43	0.000	Family is Gamma	Not Gamma
Coefficient of xbeta1 = 3	175.08	0.000	Family is Inverse Gamma	Not Inverse Gamma

Pregibon's Link Test

Dependent Variable: rawvar = ((disutility - rawyhat) ^{2})	Coeff.	P - Value
xbeta1 (Predicted Value of First Regression)	1.28^{**}	0.027
Xbeta2 = xbeta1^2	0.07	0.650
Intercept	-1.63^{***}	<0.001
Observations	2610	
Log likelihood	8130.57	
AIC	-6.22	
BIC	-12993.2	

Note: ***, ** and * indicates significance at 1%, 5% and 10% level respectively.

Testing the GLM Family of Distribution and Link Function for EQ – VAS Regression

Table 7A: GLM with Gaussian as Distribution and Log as Link

Variables		Model 1 (EQ-VAS as Dependent)	
		Coeff.	P - Value
Residence (*Ref:* Urban)	Rural	-0.01	0.443
Family Size	Number of Family Members	-0.01***	<0.001
Respondent Age	Age in Years	-0.01***	<0.001
	Age Square	0.00**	0.022
Family Income	Monthly Average	0.00***	<0.001
Marital Status (*Ref:* Otherwise)	Married	-0.02	0.179
Employment Status (*Ref:* Unemployed)	Homemaker	0.11***	<0.001
	Otherwise Employed	0.06**	0.014
Educational Attainment	Years of Education	-0.00	0.520
	Square of Education	0.00	0.149
Disease History (*Ref:* None)	Have Disease History	-0.10***	<0.001
ST Status (*Ref:* Non-User)	ST User	-0.05**	0.012
Intensity of ST Use	Number of Times Daily	-0.00	0.179
	Intensity Square	0.00	0.869
Duration of ST Use	Number of Years using ST	-0.00**	0.011
Intercept	Constant	-0.01	0.443
Observations		2610	
Log likelihood		-13893.77	
AIC		10.66	
BIC		-20264.49	

Test Regression

Dependent Variable: rawvar = ((EQVAS - rawyhat)^2)	Coeff.	P - Value
xbeta1 (Predicted Value of First Regression)	-1.76***	<0.001
Intercept	12.95***	<0.001
Log likelihood	-16583.95	
AIC	12.71	
BIC	-13511.39	

Testing Distribution Family

Hypothesis	Chi - Square	Prob.	Rule: If Do not Reject the Hypothesis then	Decision
Coefficient of xbeta1 = 0	14.94	0.000	Family is Gaussian	Gaussian
Coefficient of xbeta1 = 1	36.79	0.000	Family is Poission	Not Poission Family
Coefficient of xbeta1 = 2	68.31	0.000	Family is Gamma	Not Gamma
Coefficient of xbeta1 = 3	109.52	0.000	Family is Inverse Gamma	Not Inverse Gamma

Pregibon's Link Test

Dependent Variable: rawvar = ((EQVAS - rawyhat)^2)	Coeff.	P - Value
xbeta1 (Predicted Value of First Regression)	-22.46*	0.085
Xbeta2 = xbeta1^2	2.37	0.119
Intercept	58.12**	0.037
Log likelihood	-16568.38	
AIC	12.70	
BIC	-13534.65	

Note: ***, ** and * indicates significance at 1%, 5% and 10% level respectively.

Figure 1A: Disease Onset and ST Use

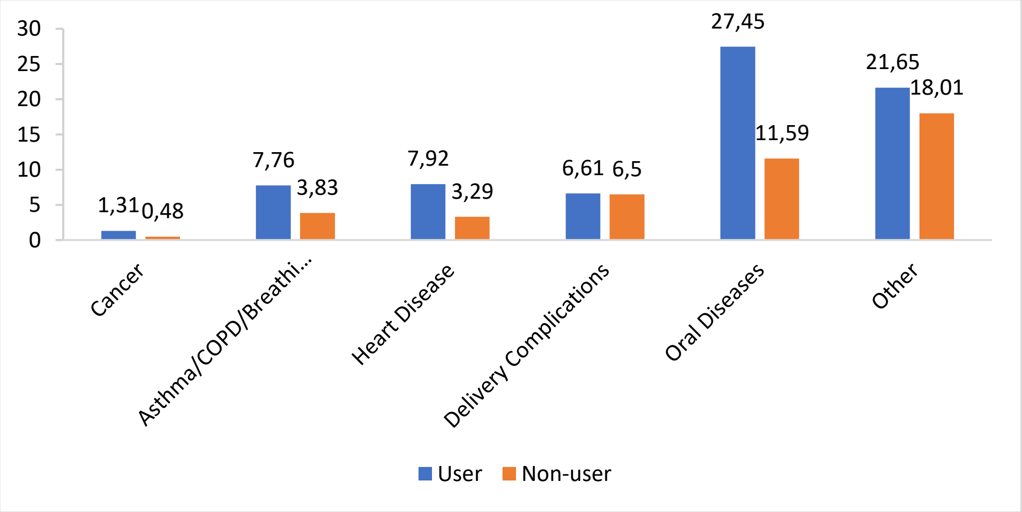

Figure 1A: Health State Comparison of SLT Users and Non - Users Employing EQ - 5D

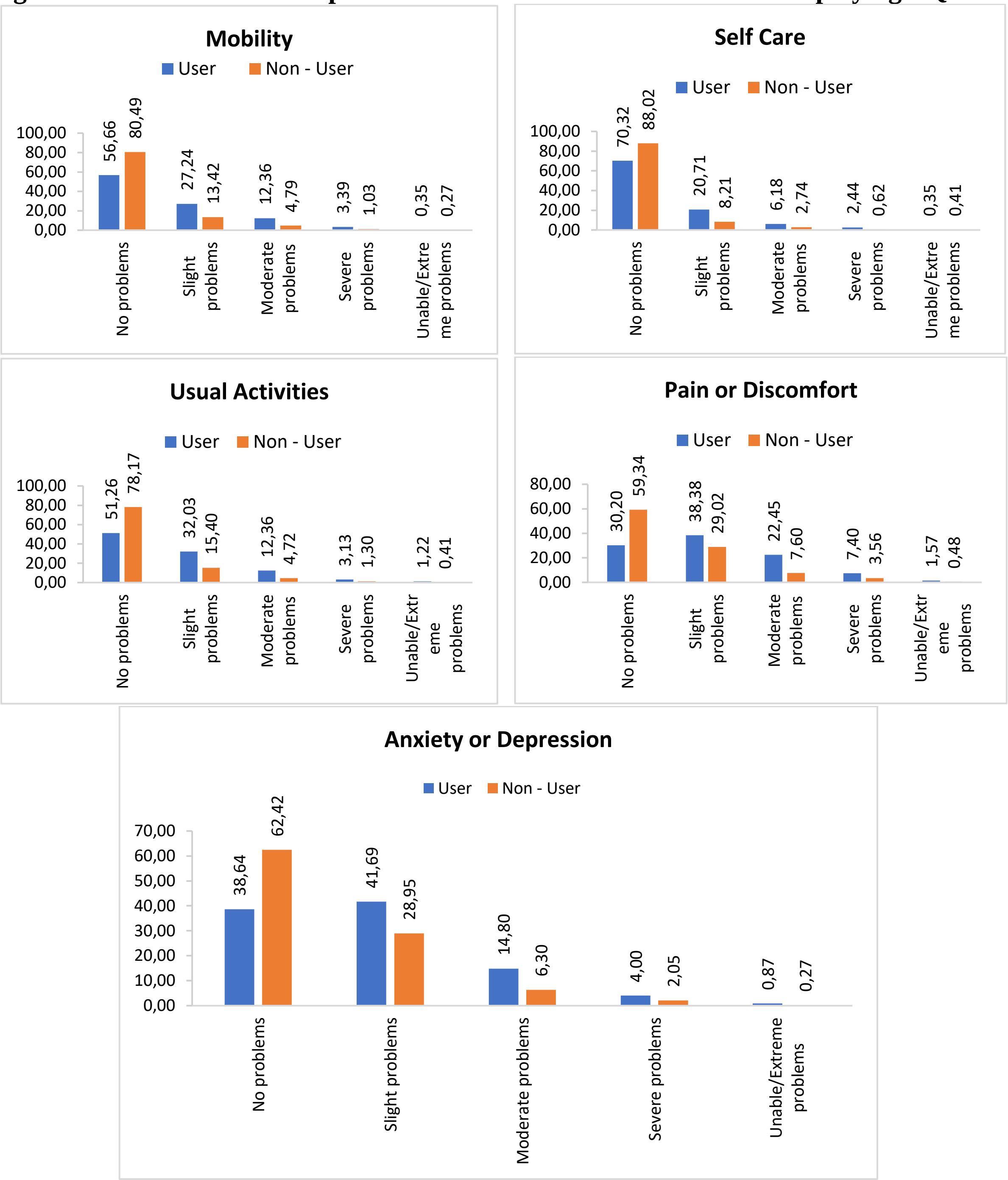

Figure 3A: Distribution of HRQoL utility score and EQ–VAS score

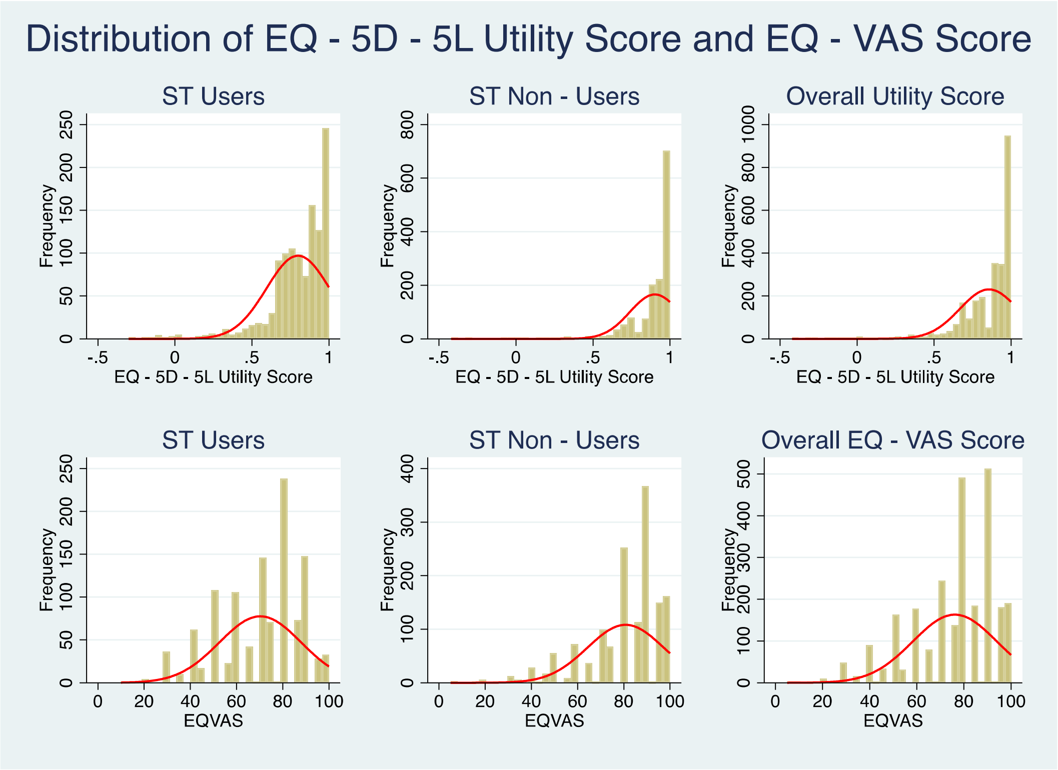

©2024 Huque R. et al.
